# Larvicidal activity of *Ocimum campechianum*, *Ocotea quixos* and *Piper aduncum* essential oils against *Aedes aegypti*

**DOI:** 10.1051/parasite/2019024

**Published:** 2019-04-17

**Authors:** Laura Scalvenzi, Matteo Radice, Luciano Toma, Francesco Severini, Daniela Boccolini, Antonino Bella, Alessandra Guerrini, Massimo Tacchini, Gianni Sacchetti, Matteo Chiurato, Roberto Romi, Marco Di Luca

**Affiliations:** 1 Department of Earth Sciences, Universidad Estatal Amazónica Km 2½ Via Puyo-Tena 160150 Puyo Ecuador; 2 Department of Life Sciences and Biotechnology (SVeB), University of Ferrara P.le Chiappini, 3, Malborghetto di Boara 44123 Ferrara Italy; 3 Department of Infectious Diseases, National Institute of Health (Istituto Superiore di Sanità, ISS) V.le Regina Elena 299 00161 Rome Italy

**Keywords:** *Ocimum campechianum*, *Ocotea quixos*, *Piper aduncum*, Essential oil, Larvicidal activity, *Aedes aegypti*

## Abstract

*Aedes aegypti*, the main arbovirus vector of the Yellow fever, Dengue, Chikungunya and Zika viruses, is widely distributed in tropical and subtropical areas throughout the world. Preventive control efforts have been implemented worldwide aimed at reducing its impact on human health. The recent reduction of chemicals available for vector control due to their negative impact on the environment and human health and the increase in mosquito resistance to insecticides have driven the research community to identify and evaluate sustainable alternatives to synthetic insecticides. In this study, the potential larvicidal effect of essential oils extracted from *Ocimum campechianum*, *Ocotea quixos* and *Piper aduncum* were tested *in vitro*. GC and GC–MS analyses showed that the main compounds were eugenol (18%), 1,8-cineole (39%) and dillapiole (48%), respectively. Susceptibility to essential oils was measured according to the WHO protocol. After 24 h, the mean percentage mortality ranged from 2.7 to 100% for *P. aduncum*, from 2.2 to 100% for *O. campechianum*, and from 2.9 to 100% for *O. quixos*. The highest potential was displayed by *P. aduncum*, followed by *O. campechianum* and *O. quixos*, with LC_50_ values of 25.7, 69.3 and 75.5 ppm, respectively. The rapid and effective larvicidal activity of these three oils led us to consider these results to be promising, also considering the possibility of local cultivation of these plants in tropical and subtropical areas and the simple technology for their large-scale preparation and production. Further studies are needed to evaluate the individual components and their activity as larvicides.

## Abstract (fr)

*Aedes aegypti*, le principal vecteur d’arbovirus qui provoque la Fièvre Jaune, la Dengue, le Chikungunya et le Zika, est largement répandu dans les régions tropicales et subtropicales du monde entier. Des efforts de contrôle préventif ont été mis en œuvre dans plusieurs pays afin de réduire son impact sur la santé humaine. La récente réduction des produits chimiques disponibles pour la lutte antivectorielle, en raison de leur impact négatif sur l’environnement et la santé humaine, et l’augmentation de la résistance des moustiques aux insecticides, ont amené la communauté des chercheurs à identifier et évaluer des alternatives durables aux insecticides synthétiques. Dans cette étude, l’effet larvicide potentiel des huiles essentielles extraites de *Ocimum campechianum*, *Ocotea quixos* et de *Piper aduncum* a été testé *in vitro*. Les analyses GC et GC-MS ont montré que les principaux composés étaient respectivement l’eugénol (18 %), le 1,8-cinéole (39 %) et le dillapiole (48 %). La sensibilité aux huiles essentielles a été mesurée conformément au protocole de l’OMS. Après 24 heures, le pourcentage moyen de mortalité variait de 2.7 à 100 % pour *P. aduncum*, de 2.2 à 100 % pour *O. campechianum* et de 2.9 à 100 % pour *O. quixos*. *P. aduncum* a affiché le potentiel le plus élevé, suivi de *O. campechianum* et de *O. quixos*, avec des valeurs CL_50_ de respectivement 25.7, 69.3 et 75.5 ppm. L’activité larvicide rapide et efficace de ces trois huiles nous a amenés à considérer les résultats comme prometteurs, compte tenu également de la possibilité de culture locale dans les zones tropicales et subtropicales de ces plantes et de la technologie simple pour leur préparation et leur production à grande échelle. Des études complémentaires sont nécessaires pour évaluer les composants individuels et leur activité en tant que larvicides.

## Introduction

*Aedes aegypti*, also named “yellow fever mosquito”, is native to Africa and now widely spread in tropical and subtropical areas throughout the world. The mosquito reached the New World from Africa by means of slave ships; casks used for shipboard storage of water must have been breeding sites for the mosquito [14]. *Ae. aegypti* is known for its relevant worldwide role as the main vector of the yellow fever virus (YFV), for which a vaccine exists, and of dengue virus (DENV), Zika virus (ZIKV), chikungunya virus (CHIKV), and other arboviruses, for which no vaccine is currently available. In particular, YFV remains a dramatic public health problem in the subtropical areas of South America and West Africa [49]. Recently, ZIKV, responsible for microcephaly, congenital nervous system malformations and Guillain-Barré syndrome has circulated in some areas of South-East Asia, tropical Africa and South America where outbreaks of infection have been well documented [50]. To take action against this mosquito vector, also able to transmit additional arboviruses heavily affecting humans, preventive control efforts are implemented worldwide and aim to reduce the impact of this mosquito species on human health.

Concerning arboviral diseases, the risk of transmission is strongly linked to vector density and for this reason, density should be rapidly reduced by vector control activities. In general, the use of synthetic insecticides has been the basis of vector control in limiting mosquito-borne diseases such as malaria, chikungunya (CHIK), dengue (DENV) and West Nile (WNV). However, over the past ten years, pesticide registration procedures aimed at minimising the negative impact of pesticides on the environment and on human health, and the widespread evolution of insecticide resistance in mosquito populations [11], have reduced the number of chemicals available for vector control [48]. As a consequence, worldwide research efforts to discover sustainable alternatives to synthetic insecticides have been implemented around plant-based pesticides that are as selective as possible toward mosquitoes [35].

Herbal extracts, linked to traditional knowledge and folk medicine, represent an almost unlimited source of bioactive compounds widely used as treatments for various human diseases [3, 7, 24] but also a source of insecticides and insect repellents. The use of plant extracts for insect control has a long historical tradition dating back 3000 years, when extracts from aromatic plants were used as repellents against ectoparasites and anthelmintics, as well as to preserve harvested foods from pests. In the 20th century, studies related to the pesticide effects of essential oils gained momentum [29]. These plant metabolites may be less sensitive to resistance development in insect populations because they are complex mixtures of numerous bioactive compounds that likely differ in their modes of action [4]. Essential oils obtained from basil (*Ocimum* spp.) have been found to be effective in repelling some species of beetles infesting foodstuffs [26]; other extracts from cumin (*Cuminum cyminum*), anise (*Pimpinella anisum*) and origan (*Origanum syriacum* var. *bevanii*) are effective against aphids and other phytophagous pests [46]. These compounds are widely used for the control of insects of health interest, such as mosquitoes.

In an effort to identify new larvicidal active ingredients (AIs) effective against *Ae. aegypti*, the potential larvicidal effects of essential oils extracted from *Ocimum campechianum* Mill. (syn. *Ocimum micranthum* Willd.), *Ocotea quixos* (Lam.) Kosterm. and *Piper aduncum* L.*,* were tested *in vitro* in this study. *O. campechianum* is a plant species in the Lamiaceae family, widespread across Central and South America. Previous studies on essential oils from *Ocimum* species showed promising results on the larvae of *Ae. aegypti* [9] and also against other insect species [18]. *O. quixos* is an evergreen tree in the Lauraceae family, native to Ecuador and Colombia. Species from the same genus, such as *O. cymbarum*, showed a remarkable mortality effect on *Ae. aegypti* larvae [40]. *P. aduncum* is an evergreen, shade-tolerant, shrubby tree in the Piperaceae family, native to the West Indies and tropical America. Essential oils from the *Piper* genus are recognised as having insecticidal effects against a large number of insects. In particular, *P. aduncum* essential oil recorded very good activity against the *Ae. aegypti* mosquito [25].

These three plant species were selected because they belong to genera with promising chemical characteristics when used as insecticides, and because they are from Ecuador, which belongs to a selected group of 17 countries defined as “Megadiverse” due to their impressive biological diversity [21, 43], an important source of possible bioactive compounds. Essential oils from the Ecuadorian Amazon Region have been investigated in the last few decades in order to deepen our understanding of their biological activities [9, 19, 38, 39, 45]. To our knowledge, studies evaluating the larvicidal effects of essential oils from Ecuadorian plants against *Ae. aegypti* have not been reported to date.

## Materials and methods

### Plant material and extraction of essential oils

Aerial parts of *O. campechianum, O. quixos* and *P. aduncum* were collected from a wild population in the Amazonian region of Pastaza (Ecuador). Species authentication was certified by Dr. David Neill and voucher specimens from each plant were deposited at the Herbarium ECUAMZ of the Amazonian State University (UEA) in Ecuador (voucher specimen: Radice 18070D, Neill 18070B, Scalvenzi 18070C).

The essential oils were obtained by hydrodistillation in a stainless steel distiller equipped with a Clevenger apparatus. All essential oils were obtained performing three distinct distillations and essential oil (moisture-free) yield was 0.68% for *O. micranthum*, 0.13% for *O. quixos*, and 0.12% for *P. aduncum*, respectively. The oil was dried over anhydrous sodium sulphate and stored in sealed amber vials at 4 °C.

### GC and GC/MS analyses

Compound identification was carried out by gas chromatography and gas chromatography – mass spectrometry (GC and GC–MS) analyses, and the relative peak areas for individual compounds were averaged. For the analysis, a ThermoQuest GC-Trace gas chromatograph equipped with a FID detector and a Varian FactorFour VF-5 ms poly-5% phenyl-95%-dimethylsiloxane column (30 m × 0.25 mm i.d., film thickness: 0.15 μm) were used. Operating conditions were as follows: injector temperature 250 °C, FID temperature 250 °C, carrier (Helium) flow rate 1 mL/min, and split ratio 1/20. The initial oven temperature was 55 °C and then increased to 100 °C at a rate of 1 °C/min, then to 250 °C at a rate of 5 °C/min and then kept constant at 250 °C for 15 min. One microliter for each replicate was dissolved in CH_2_Cl_2_ and injected. The oil percentage composition was computed by the normalisation method from the GC peak areas, without using correction factors. The chemical characterisation of essential oil compounds was performed by a Varian GC-3800 gas chromatograph equipped with a Varian MS-4000 mass spectrometer using electron impact and hooked to NIST 05 (National Institute of Standards and Technology) Mass Spectral Library. The conditions were the same as those described for GC analysis and the same column was also used. The mass spectroscopy conditions were as follows: ionisation voltage, 70 eV; emission current, 10 μAmp; scan rate, 1 scan/s; mass range, 29–400 Da; trap temperature, 150 °C, transfer line temperature, 300 °C. The essential oil compounds were characterised by comparing their relative retention times, KI, and the MS fragmentation pattern with those of other known essential oils, with pure compounds and by matching the MS fragmentation patterns and retention indices with the above-mentioned mass spectra libraries and with those in the literature [2]. The Kovats index of the components was determined adding a C_8_–C_32_ *n*-alkanes (Sigma-Aldrich) to the essential oil before injecting in the GC–MS equipment and analysed under the same conditions reported above [19].

The local name and main essential oil compounds are shown in Table 1. For the larvicidal assays, the essential oil was dissolved in a solution of 1% dimethyl sulfoxide (DMSO) in test medium.

**Table 1:** Chemical characterisation of essential oil from leaves of *Ocimum campechianum*.

No.	Component^a^	Area%^b^	RI exp^c^	RI lit^d^
1	α-pinene	0.7	929	932
2	camphene	0.1	944	946
3	sabinene	0.3	967	969
4	β-pinene	1.2	973	974
5	myrcene	0.3	987	988
6	p-cymene	0.5	1021	1020
7	o-cymene	0.4	1025	1023
8	1,8-cineole	11.4	1028	1026
9	*cis*-ocimene	8.0	1032	1032
10	linalool	2.9	1101	1095
11	*allo*-ocimene	0.1	1126	1128
12	α-terpineol	0.4	1193	1186
13	δ-elemene	0.6	1337	1335
14	eugenol	18.6	1363	1356
15	α-copaene	0.5	1376	1374
16	elemene isomer	0.4	1383	–
17	β-elemene	8.9	1388	1389
18	β-caryophyllene	17.0	1410	1416
19	γ-elemene	0.5	1427	1434
20	*trans*-α-bergamotene	0.4	1431	1435
21	α-caryophyllene	4.5	1451	1452
22	*allo*-aromadendrene	1.5	1455	1458
23	germacrene D	0.4	1477	1484
24	β-selinene	1.8	1484	1489
25	viridiflorene	0.2	1489	1696
26	bicyclogermacrene	9.0	1490	1500
27	germacrene A	1.4	1500	1508
28	germacrene B	1.4	1556	1559
29	spathulenol	1.9	1576	1577
30	caryophyllene oxide	1.7	1581	1582
	Total identified	97.2		

a Components are listed in order of elution and their nomenclature is in accordance with the NIST 05 (National Institute of Standards and Technology) Mass Spectral Library.

b Relative peak areas, calculated by GC-FID.

c RI exp: linear retention indices calculated on a VF-5 MS column.

d RI lit: linear retention indices [2].

### Larvicidal assay

A long-established laboratory colony of *Ae. aegypti* (collected in Reynosa, Mexico, in 1998) was used as a susceptible strain for larvicidal bioassays. To allow hatching, eggs were placed in plastic trays (30 × 15 × l0 cm) containing 1 L of dechlorinated tap water. The larvae were reared in a climatic chamber at a temperature of 27 ± 1 °C, 80 ± 10% relative humidity, and a photoperiod of 14:10 h light:dark, until reaching adulthood. The bioassays were performed according to the procedure of the World Health Organization [47]. Specifically, every bioassay was carried out in a climatic chamber with the above-reported temperature and photoperiod and replicated three times with mosquitoes from different rearing batches.

Each essential oil was dissolved in dimethylsulphoxide (DMSO) to prepare graded concentrations of tested material. Tests consisted of at least three replicates per concentration and were performed by serial dilutions of each oil (1000, 500, 250, 100, 75, 50, 37.5, 25, and 12.5 μg/mL). Batches of 25 late 3rd/early 4th-stage larvae were exposed to different doses of each oil in plastic cups, each containing 250 mL of dechlorinated water. Over 8000 larvae were tested (a mean of 2700 per oil). After 24 h of exposure, during which no food was given to the larvae, mortality was checked (considering both dead and moribund larvae), and expressed as percentage mortalities. Larvae were considered moribund or dead when they showed unnatural positions, tremors, in coordination, or rigor; they did not respond to stimuli such as probing with a needle; or they were incapable of rising to the surface of the water.

Negative and positive control tests were performed in parallel for comparison. The first negative control contained ordinary laboratory larval rearing water; the second negative control contained an aqueous solution of 0.88% of dimethylsulfoxide (DMSO), used as a dispersing medium for the essential oils. The positive control contained an aqueous solution 0.05 mg/L of the organothiophosphate insecticide Fenthion (according to the diagnostic dosages for control of *Ae. aegypti*, indicated by the World Health Organization [47].

### Statistical analysis

A log-probit regression model for the three oils was obtained and toxicity was reported as lethal concentrations LC_50_ and LC_90_, representing the concentration in ppm necessary to cause 50% and 90% larval mortality, respectively, in 24 h. The larvicidal mortality was corrected by Abbott’s formula [1] and an LC_50_, LC_90_ regression equation, and the 95% confidence limit was calculated by using probit analysis [17]. Probit analysis was performed by using the software by Dr. Alpha Raj. M “Free LD_50_/LC_50_ Calculator (2018 web version)” based on the Probit Analysis method of Finney [16].

## Results

### Chemical characterisation of essential oils

The compounds of *O. campechianum* essential oil are reported in Table 1; distillation yields were of 0.68%. As previously published by us [34], the yields for *O. quixos* essential oil were 0.13%, for *P. aduncum* 0.12%. The essential oil of *O. campechianum* was found to be rich in eugenol (18.6%), β-caryophyllene (17.0%), 1,8-cineole (11.4%), bicyclogermacrene (9%), *cis*-ocimene (8%) and α-caryophyllene (4.5%), that of *O. quixos* was characterised by 1,8-cineole (39.2%), sabinene (6.5%), α-pinene (6.3%), β-caryophyllene (4.7%) and terpinen-4-ol (4.2%), and *P. aduncum* essential oil showed a high percentage of dillapiole (48.2%) followed by *trans*-ocimene (7.5%) and β-caryophyllene (4.8%) [34].

### Larvicidal bioassay

**Figure 1:**
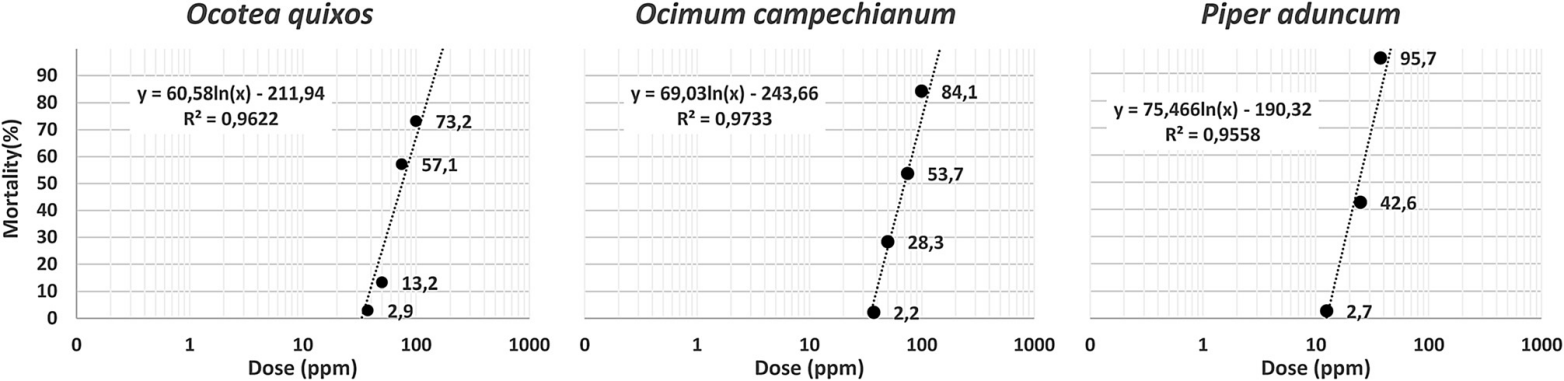
Log-probit regression lines from the analysis of the collected data about mortality rates in *Ae. aegypti*, for each tested essential oil.

From the range of doses used, mortality was found to be dose-dependent. After 24 h, the mean percentage mortality ranged from 2.7 to 100% for *P. aduncum*, from 2.2 to 100% for *O. campechianum*, and from 2.9 to 100% for *O. quixos*. The interval observations in each concentration among oils showed the highest activity responses after 24 h. The highest potential was displayed by *P. aduncum*, followed by *O. micranthum* and *O. quixos*, with an LC_50_ of 25.73, 69.29 and 75.51 ppm, respectively (Table 2, Fig. 1).

**Table 2:** Larvicidal effect of *Piper aduncum, Ocimum campechianum* and *Ocotea quixos* essential oils on *Aedes aegypti* at different concentrations, after 24 h of exposure, expressed as percentage of mortality.

EO^a^	Time (h)	Concentration									LC_50_ b (CI_95_)^c^	LC_90_ b (CI_95_)^c^	LC_99_ b (CI_95_) c	DF^d^	SLOPE^e^	*v*^2^
		1000	500	250	100	75	50	37.5	25	12.5						
*P. aduncum*	24	100	100	100	100	100	100	95.7	42.6	2.7	23.73 (19.62–28.69)	35.51 (29.36–42.93)	49.31 (40.78–59.63)	2	7.43	0.003
*O. campechianum*	24	100	100	100	84.1	53.7	28.3	2.2	0	0	69.29 (58.69–81.81)	109.46 (92.71–129.24)	158.91 (134.59–187.63)	2	1.47	0.000
*O. quixos*	24	100	100	100	73.2	57.1	13.2	2.9	0	0	75.51 (63.22–90.19)	122.56 (102.6–146.39)	181.89 (152.28–217.26)	2	6.10	0.188

a EO: essential oil.

b LC: lethal concentrations.

c CI: confidence intervals.

d DF: dialysable fraction.

e SLOPE: slope of regression line, *p*-value, 95%.

### Statistical analysis

Regression models were determined from a minimum of three to a maximum of four points, giving a rate of mortality comprised between 2.2 and 95.7%. All of the tests reported in this study provided regression lines with values of *χ* ^2^ < 5, degree of freedom > 1, and slope (from 0.54 to 0.8).

## Discussion and conclusion

### Essential oils

The essential oil of *O. campechianum* was found to be rich mainly in eugenol (18.6%) and β-caryophyllene (17.0%), which is consistent with results reported by other authors [22, 38]. The constituents of the essential oil obtained from *O. quixos* showed 1,8-cineole as the most abundant compound (39.2%), confirming previous studies carried out in Ecuador [24]. Sabinene (6.5%) and α-pinene (6.3%) were also found to be relevant compounds in other research [36, 41]. Nevertheless, the most characteristic compounds such as *trans*-cinnamaldehyde and β-caryophyllene were not found in the essential oil investigated in the present research. It is known that essential oils from the *Ocotea* genus usually show an high degree of chemodiversity, as reported in previous studies [33, 39], and this could explain its unusually composition. The essential oil of *P. aduncum* was mainly characterised by dillapiole (48.2%), *trans*-ocimene (7.5%) and β-caryophyllene (17.0%), which is in line with other studies carried out on Ecuadorian *Piper* species [18].

Concerning the essential oil (EO) yields, it is important to point out that even though the distillation yields were low, the implementation of such EOs is feasible, due to the easy reproduction of the studied species, both on a spontaneous and human-mediated basis. Moreover, at least in the case of *O. campechianum*, studies on elicitation techniques have been reported for similar species (*O. basilicum* L.), showing promising results concerning the significant increase in total essential oil amount, using methyl salicylate, methyl jasmonate and chitosan as elicitors [15].

### Anti-larval effects of essential oils

The present study shows findings of *Ae. aegypti* larvicidal activity of three essential oils extracted from the leaves of *P. aduncum*, *O. campechianum*, and *O. quixos* from Ecuador. These encouraging results should stimulate the search for additional selective, eco-friendly larvicidal compounds from Ecuador, where the flora exhibits a richness in diversity of aromatic plants with potential as natural extracts for control of mosquito vectors.

Previous studies on *Piper* spp*.* have recognised that essential oils from plants belonging to this genus are very effective as botanical insecticides against several arthropods, including mosquitoes. *Piper tuberculatum* and *Piper aduncum* extracts were highly effective against larvae and engorged female *Rhipicephalus microplus* ticks [13, 44]. The essential oil of *P. nigrum* exhibited insecticidal activity against the mosquito species *Ae. aegypti*, *Anopheles stephensi* and *Culex quinquefasciatus* [28]. A study performed in the Brazilian Amazon comparing the methanol leaf and root extracts from several local plants, at the concentration of 500 μg/mL (500 ppm), reported *P. aduncum* to have very good activity against *Ae. aegypti*, showing 100% mortality after 24 h, similarly to our results, because of the phytochemical compound dillapiole [32]. Previous studies indicate a relevant potential for dillapiole to be used as a synergist compound in combination with pyrethroid-based insecticides to control *Ae. aegypti* [28].

*O. campechianum* was already used as an insecticide against white fly *Aleurodicus cocois* (Curtis, 1846) (Hemiptera, Aleyrodidae) [22]. Researchers carried out a study testing essential oils from *O. americanum* and *O. gratissimum* against larvae of *Ae. aegypti* and obtained LC_50_ values of 67 ppm and 60 ppm respectively, values very close to the 69.29 ppm of *O. campechianum* found here [10]. The literature indicates that eugenol, the main compound of *O. campechianum* essential oil studied in this research, exhibits larvicidal activity against *Ae. aegypti* at the concentration of 44.5 ppm. Therefore, the larvicidal activity of *O. campechianum* essential oil against this mosquito species could be highly associated with the presence of eugenol [6].

Concerning the essential oil of *O. quixos*, it has rarely been reported for its possible use against mosquito larvae as it is used mainly as a disinfectant, local anaesthetic, and anti-diarrheic infusion [5]. However, the results reported here about *O. quixos* (LD99 = 181.89 ppm) are consistent with findings on *Ocotea cymbarum*, showing that its strongest toxic effect was 100% mortality in *Ae. aegypti* third age larvae at concentrations ≥30 ppm [23]. Previous studies on the monoterpene 1,8-cineole, which is the main constituent of *O. quixos* oil in this experiment, showed larvicidal activity toward the larvae of several mosquitos including *Ae. aegypti* and *Ae. albopictus*. The hydrophobicity of 1,8-cineole is probably the main factor responsible for toxicity towards larvae, because cuticle penetration is facilitated [12].

The larvicidal activity of all three biological compounds was evaluated at 24 h after treatment and it was found to be high for all of the three oils.

However, it should be noted that costly and lengthy authorisation processes and the low environmental persistence of essential oils (and thus their effects) strongly limit the availability on the market of commercial insecticidal and repellent products. In addition, botanical larvicides (Bls) based on EOs may exhibit different efficacy depending on the post-application temperature of the environment, and this effect seems to be related to the non-uniform behaviour of their major constituents [30]. In order to provide clear and practical recommendations for application of such BIs, it is fundamental to understand the relationship between post-application temperature and the insecticidal efficacy of EOs. Therefore, further studies to investigate this aspect are needed.

An additional aspect that markedly limits the development of such products for real use is the undeniable lack of field studies validating the efficacy of herbal extracts as mosquito larvicides. Nevertheless, some promising results regarding field experiments have been reported in a recent review article concerning the application of herbal extracts as mosquito larvicides [31]. Herein, at least 14 research studies are mentioned as showing preliminary and encouraging results on the treatment of external water storage reservoirs or sewage water bodies as typical breeding sites for *Ae. aegypti, Ae. albopictus, Cx. quinquefasciatus* and *An. stephensi.*

Clearly, several biological insecticides based on Spinosad or *Bacillus thuringiensis* var. *israelensis* (B.t.i.), currently used for mosquito control, show greater efficacy than the oils tested in this study and in others. According to Bond et al. and Romi et al. [8, 37], the larvicidal activity of Spinosad ranged between 0.01 and 0.02 ppm (LC_50_ and LC_99_ respectively at 24 h after treatment), and similar larvicidal activity was reported for B.t.i [20].

For completeness of comparison, we report the susceptibility of *Ae. aegypti* to the organothiophosphate insecticide Fenthion, used as a positive control in this study, as a lethal dose of 0.05 ppm, corresponding to the LC_99_ for this species [47].

Finally, our results show clear larvicidal activity against *Ae. aegypti* for all three essences, with *P. aduncum* essential oil displaying a greater efficacy when compared to *O. campechianum* and *O. quixos* oils.

Further studies are of course needed to understand the effectiveness of the various components present in each extracted oil and the mechanisms of action that are at the basis of their larvicidal activity against *Ae. aegypti*.

In conclusion, the rapid and effective larvicidal activity of the three oils led us to consider results to be promising, considering that these plants are commonly widespread as wild species all over the Ecuadorian Amazon Region. In addition, they could also be easily cultivated on human-controlled systems, both locally and in other tropical countries. Reproduction of *O. campechianum* plants usually occurs through spontaneous seed dissemination, while *O. quixos* and *P. aduncum* reproduce through vegetative processes. Lastly, essential oils from these plant species can be prepared using simple technology.
